# Digoxin Derivative BD-8 Attenuates Oxidative Stress and Neuroinflammation in an LPS-Induced Experimental Model

**DOI:** 10.1007/s12013-026-02108-x

**Published:** 2026-06-25

**Authors:** Vitória Karolina Brandão Souza, Davi Azevedo Ferreira, Antônio Pereira Ribeiro Arantes, Elton de Sá Figueiredo, Millena Ferreira Rodrigues Fonseca, Pâmela Yasmin de Oliveira Ferreira, Martina Raissa Ribeiro, Paulo César Ghedini, Andreza Amália de Freitas Ribeiro, José Augusto Ferreira Perez Villar, Maira de Castro Lima, Israel José Pereira Garcia, Hérica L Santos, Cristoforo Scavone, Sandra Rodrigues Mascarenhas, Jacqueline Alves Leite

**Affiliations:** 1 Instituto de Ciências Biológicas, Department of Pharmacology, Federal University of Goiás, Goiânia, 74690-612 GO Brazil; 2 Laboratory of Immunopharmacology, Biotechnology Center, Federal University of Paraíba, PB João Pessoa, Brazil; 3 Laboratório de Bioquímica Celular, Federal University of São João del-Rei, Campus Centro-Oeste Dona Lindú, Divinópolis, MG Brazil; 4 Neuropharmacology Research Laboratory, University of São Paulo, São Paulo, 05508-000 SP Brazil; 5 Laboratório de Síntese Orgânica e Nanoestruturas – Campus Centro-Oeste Dona Lindú, Federal University of São João del-Rei, Divinópolis, MG Brazil

**Keywords:** Na⁺, K⁺-ATPase, BD-8, Oxidative stress, Cardiosteroids, Neuroprotection

## Abstract

Chronic neuroinflammation contributes to neuronal damage through oxidative stress and inflammatory mediators, highlighting the need for novel therapeutic strategies. This study evaluated the cytotoxicity and neuroprotective effects of BD-8, a digoxin-derived cardiosteroid, in vitro and in vivo. BD-8 showed lower cytotoxicity than digoxin, with CC₅₀ values of 7.4 µM in SH-SY5Y cells and 8.5 µM in BV-2 cells, while digoxin presented a CC₅₀ of 33 nM in SH-SY5Y cells. In vivo, BD-8 did not alter cardiac, hepatic, or renal biochemical markers, supporting its systemic safety profile. In the open-field test, BD-8 prevented LPS-induced locomotor impairment, significantly increasing total crossings at 0.56 mg/kg (62.07 ± 3.269; *P* < 0.05) and 1.12 mg/kg (66.31 ± 5.282; *P* < 0.001) compared to the LPS group. BD-8 also reduced lipid peroxidation levels in the cortex at 0.56 mg/kg (14.38 ± 0.5568; *P* < 0.001) and 1.12 mg/kg (13.94 ± 0.8372; *P* < 0.001), and in the hippocampus at 1.12 mg/kg (14.04 ± 0.5223; *P* < 0.001). Additionally, BD-8 modulated antioxidant enzyme activities, superoxide dismutase (SOD), catalase (CAT), glutathione peroxidase (GPx), in both the cortex and hippocampus and reduced IL-6 levels in these brain regions without altering BDNF (Brain Derived Neurotrophic Factor) levels. These findings support BD-8 as a promising candidate for neuroinflammatory disorders.

**Supplementary Information:**

The online version contains supplementary material available at https://doi.org/10.1007/s12013-026-02108-x.

## Introduction

Peripheral inflammation, whether due to viral infections, autoimmune diseases, or acute conditions such as sepsis, can trigger neuroinflammation [1]. This inflammatory response in the brain can become chronic, characterized by the activation and persistence of microglia and astrocytes, and recent evidence indicates that inflammation is not only a consequence but a significant etiological factor in the progression of neurodegenerative diseases [2]. Among the mechanisms involved in this process, oxidative stress plays a central role and is characterized by an imbalance between the production of reactive oxygen species (ROS) and the antioxidant defense capacity of cells. Due to its high metabolic demand and oxygen consumption, the brain is particularly susceptible to oxidative damage, which may lead to lipid peroxidation, protein oxidation, mitochondrial dysfunction, and neuronal death [3, 4].

Lipopolysaccharide (LPS), a bacterial endotoxin, is widely used as an experimental model of neuroinflammation because it induces ROS production, neurotoxicity, synaptic dysfunction, and behavioral alterations associated with sickness behavior and depressive-like symptoms [5, 6]. Therefore, the LPS model has been extensively employed to investigate the mechanisms underlying neuroinflammation and to evaluate potential anti-inflammatory and antioxidant therapies targeting the CNS.

Cardiotonic steroids (CTSs) are classical ligands of Na⁺/K⁺-ATPase (NKA), an enzyme responsible for maintaining cellular electrochemical gradients through ATP hydrolysis, thereby supporting neuronal excitability and synaptic transmission [7, 8]. Beyond its canonical ionic function, NKA also acts as a signaling platform capable of activating pathways involved in cell survival, inflammation, and oxidative stress modulation. Previous studies demonstrated that CTSs, at nanomolar concentrations, can activate NKA-mediated signaling pathways and exert neuroprotective effects in models of ischemia and neurodegeneration [9, 10].

Endogenous cardiotonic steroids, such as Ouabain and digoxin, are classic ligands [11] of Na⁺/K⁺-ATPase and exhibit anti-inflammatory effects [12]. These compounds modulate processes such as cell migration, vascular permeability, oxidative stress, and the production of pro-inflammatory cytokines [13–17]. Our group has demonstrated that Ouabain reduces pro-inflammatory cytokines and inhibits the MAPK p38 and nuclear translocation of nuclear factor kappa B (NF-κB) signaling pathways [18] (Cavalcante-Silva et al., 2021), in addition to attenuating characteristics of the allergic asthmatic response [19] (Galvão et al., 2022).

Digoxin, in turn, appears to inhibit the differentiation of Th0 cells into Th17 cells [11, 20, 21]. The neuroprotective activity of Ouabain has also been described [22]. In vivo studies show that it exerts a protective effect against LPS-induced neuroinflammation in the hippocampus of rats, reducing the activation and NF-κB, thereby decreasing the expression of iNOS and IL-1β [23]. In addition, Ouabain reduced oxidative stress in the cerebellum of LPS-challenged rats [24]. Additionally, digoxin significantly rescued rats from memory loss caused by intracerebroventricular injection of streptozotocin, decreasing hippocampal cell death, neuroinflammation, and cholinergic deficiency [25].

Our group developed new digoxin derivatives that explore α-NKA specificity for their therapeutic potential [26]. The compound 21BD exhibits distinct chemical properties but maintains significant anti-inflammatory effects [27]. Another derivative, BD-15, demonstrated neuroprotective actions, including inhibition of H₂O₂ production, lipid peroxidation, and other markers of oxidative stress, reinforcing the potential of digoxin derivatives as therapeutic agents [28, 29]. More recently, our team developed the derivative BD-8, which selectively stimulated the α2 isoform of Na⁺/K⁺-ATPase in a heterologous Sf9 system [30]. Our data demonstrated that BD-8 decreases the expression of iNOS and the phosphorylation of NF-κB p65, ERK, and p38 in primary macrophage culture [31]. Furthermore, BD-8 showed anti-inflammatory activity in vivo in a carrageenan-induced paw edema model in mice [31]. However, despite its promising anti-inflammatory profile, the effects of BD-8 in the CNS remain unknown. Based on the established role of oxidative stress and neuroinflammation in LPS-induced brain injury, as well as the previously described pharmacological properties of CTS derivatives, we hypothesized that BD-8 could exert neuroprotective effects in the CNS. Therefore, this study aimed to investigate the effects of the digoxin derivative BD-8 in an experimental model of LPS-induced neuroinflammation and oxidative stress.

## Materials and Methods

### Chemistry

The digoxin derivative (BD-8) was synthesized via a vinylogous aldol reaction followed by rearrangement, resulting from the chemical modification of the digoxin molecule with an appropriate benzaldehyde [30, 31]. The structure shown here corresponds to a revised version of the structure previously reported (see supporting information). The results of this new structural revision will be communicated in detail in a forthcoming study. This synthesis was carried out by the Laboratory of Organic Synthesis and Nanostructure of the Federal University of São João del-Rei.

**Figure:**
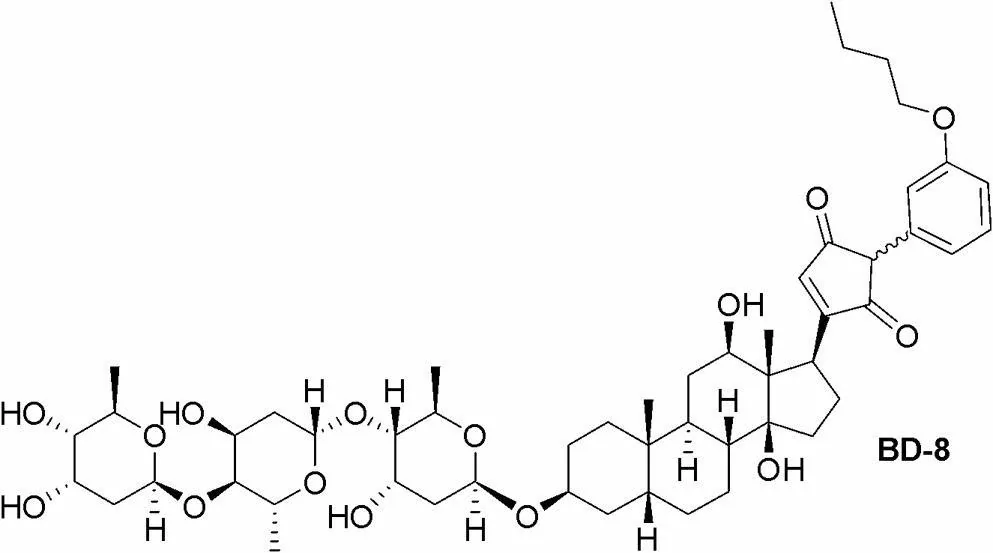

### Cell Lines

SH-SY5Y cells were acquired from the American Type Culture Collection located in Manassas, Virginia. They were cultivated in a mixture of DMEM and Ham’s F-12 media provided (Gibco, Carlsbad, CA), enhanced with 10% fetal bovine serum (Bionutrientes), penicillin/streptomycin (Gibco) with concentration 100 U/mL, and 1 mM sodium pyruvate (Sigma), all while incubating at 37 °C with 5% CO2. The BV-2 cell line, which is derived from C57BL/6 mouse brain microglia and has the BCRJ reference number 0356, was maintained in RPMI-1640 medium that included L-Glutamine, sodium bicarbonate, and 10% FBS, with a temperature set at 37 °C and 5% CO_2_.

### Cell Viability Assay

The cytotoxicity of BD-8 (Sigma-Aldrich, O3125) was evaluated using an MTT (3-[4,5-dimethylthiazol-2-yl]-2,5-diphenyltetrazolium bromide) method (Sigma). In summary, SH-SY5Y and BV-2 cells were placed into 96-well plates (4 × 10⁴ cells per well for SH-SY5Y and 1 × 10⁴ cells per well for BV-2) to achieve an 80–90% confluence within 24 h, followed by treatment with various concentrations of BD-8. After 120 h, MTT (1 mg/mL) was introduced into each well, and the plates were incubated for 4 h at a temperature of 37 °C with 5% CO2. Formazan crystals were dissolved, and the optical density was measured using spectrophotometry at a wavelength of 570 nm.

### Animals

The experiments related to toxicology were conducted using male Wistar rats that were two months old, sourced from the UFSJ Central Animal Facility and housed in the CCO Sectorial Animal Facility. The environment maintained a controlled temperature of 22 degrees Celsius, with a variation of plus or minus two degrees, alongside a light/dark cycle lasting 12 h, allowing the animals free access to food and water.

For the evaluation of neuroprotection involving BD-8, male Swiss mice aged two months were sourced from the Central Animal Facility at the Federal University of Goiás (UFG). The mice were housed in an environment with a regulated temperature of 22 °C ± 2 °C, maintained under a 12-hour cycle of light and darkness, and had unrestricted access to both food and water.

### Toxicological Test

The rats were categorized into five distinct groups: Control (DMSO; 0.1%), BD-8 (0.02 mg/kg), BD-8 (0.1 mg/kg), BD-8 (0.2 mg/kg), and BD-8 (0.4 mg/kg). The subjects received intraperitoneal (i.p.) administration for three consecutive days. On the final day of treatment, the open field assessment was conducted. Seventy-two hours following the treatment, the rats were sacrificed using a combination of Ketamine (180 mg/kg) and Xylazine (30 mg/kg). The hearts were collected and stored at -80 °C for subsequent determination of Na/K-ATPase activity and oxidative stress evaluation. The detailed methodologies for these techniques are provided in the Supplementary Material.

### Neuroprotective Activity

In this research, the subjects were randomly divided into five categories (*n* = 8): Control (DMSO; 0.1%), LPS (1 mg/kg) (Escherichia coli serotype O111:B4), BD8 (0.28 mg/kg), BD8 (0.56 mg/kg), and BD8 (1.12 mg/kg). After one hour, the subjects were exposed to LPS (1 mg/kg, injected into the peritoneum), except for the control group, which was given a saline solution (0.9%). Four hours following the exposure, the open field assessment was conducted to observe sickness-related behaviors. A day after the LPS treatment, the subjects were euthanized, and samples from the total cortex and hippocampus were gathered for biochemical examinations.

### Open Field

The open-field assessment was conducted to assess the toxicological impact on rats and to study the movement and exploration behavior in mice. For the toxicological assessment, the animals were housed in an acrylic enclosure (70 cm × 70 cm × 40 cm) for a duration of 5 min. The parameters observed included: the frequency of shifts between squares, the frequency of transitions to the central square, and the instances of the animals lifting their front paws, which were utilized to gauge their movement and exploration activities [32]. To evaluate behavioral alterations after LPS injection, mice were placed individually in a round arena (300 × 300 mm) and monitored for 5 min. The overall number of crossings into different quadrants and the duration spent in the central area were noted.

### Plasma Analysis

Blood samples were obtained from anesthetized rats via intracardiac puncture. The samples were then centrifuged at 1,000 g for 15 min at a temperature of 4 °C. The resulting plasma was frozen at -80 °C for future biochemical assessments. A commercial kit was used to analyze total proteins, glucose levels, total cholesterol, total triglycerides, total lactate, total bilirubin, AST, ALT, CK-MB, and lactate dehydrogenase (LDH), following the guidelines provided by the manufacturer (Labtest, Bioclin, Analisa).

### Preparation of Brain Samples and Protein Content Determination

After the last behavioral assessment, the subjects were sedated via intraperitoneal injection of ketamine combined with xylazine hydrochloride. Following this, they were put down through decapitation, and both the hippocampus and entire cortex were meticulously removed and separated. The tissue samples were blended in a solution of 50 mM potassium phosphate buffer at a pH of 7.4 in a ratio of 1:6 (weight/volume). The homogenate obtained was centrifuged at 8,000 × g for 10 min to separate the low-speed supernatant. The overall protein concentration in the samples was analyzed utilizing the Bradford assay [33], employing bovine serum albumin (BSA) as a reference standard.

### Lipid peroxidation (LPO) Levels

Thiobarbituric acid-reactive substances (TBARS) were assessed using the technique outlined by Ohkawa, Ohishi, and Yagi in 1979, with slight adjustments. For this analysis, a solution containing 10% trichloroacetic acid (TCA), 0.8% thiobarbituric acid (TBA), and 3% sodium dodecyl sulfate (SDS) was incorporated into the HT fraction. This mixture was subjected to heating at 95 degrees Celsius for one hour. The resultant product from the reaction was measured using a spectrophotometer at a wavelength of 532 nanometers. The findings were reported as malondialdehyde (MDA) equivalents, which were derived from a standard curve of MDA and presented in terms of nmol per mg of protein.

### Superoxide dismutase (SOD) Activity

The activity of SOD was determined spectrophotometrically using a slightly modified method derived from the approach of Misra and Fridovich (1972) [34]. This method is based on SOD’s ability to inhibit the autoxidation of adrenaline. Specifically, the S1 fraction was incubated with 60 mM adrenaline tartrate and glycine buffer (50 mM, pH 10). The absorbance was measured at 480 nm. Enzyme activity was expressed as SOD units (U) per milligram of protein.

### Catalase (CAT) Activity

CAT activity was determined spectrophotometrically by measuring the decomposition of H₂O₂, following a slightly modified method based on Aebi’s (1984) [35] approach. Specifically, the S1 fraction was incubated with 86 mM H₂O₂ and 50 mM sodium phosphate buffer (pH 7.0). The reaction progress was monitored using a spectrophotometer at 240 nm. Enzyme activity was expressed as CAT units (U) per milligram of protein.

### Glutathione peroxidase (GPx) Activity

The activity of GPx was determined using a slightly modified method described by Wendel (1981) [36]. Specifically, the S1 fraction was placed in a microplate containing 100 mM potassium phosphate buffer and 90 mM sodium azide and incubated at room temperature in the dark for 30 min. After incubation, 100 mM reduced glutathione (GSH), 20 mM NADPH, and 2 U/mL glutathione reductase (GR) were added. The reaction was initiated with 100 mM H₂O₂, and the decrease in absorbance due to NADPH oxidation was measured at 340 nm. GPx activity was expressed as U/mg protein.

### Protein Extraction and Quantification

Cortical and hippocampal tissue samples were homogenized, and total proteins were extracted using RIPA lysis buffer according to a protocol adapted from Harlow and Lane. Protein concentrations in the resulting lysates were determined by the Bradford assay using a protein quantification kit from Bio-Rad Laboratories.

### Quantification of IL-6 and BDNF by ELISA

The levels of interleukin-6 (IL-6) and brain-derived neurotrophic factor (BDNF) in cortical and hippocampal tissues were quantified by enzyme-linked immunosorbent assay (ELISA), according to the manufacturer’s instructions. IL-6 concentrations were determined using the mouse IL-6 DuoSet ELISA kit (Cat. No. DY406-05), and BDNF levels were measured using the mouse BDNF DuoSet ELISA kit (Cat. No. DY248), both obtained from R&D Systems. Absorbance was measured using a microplate reader at 450 nm with wavelength correction at 570 nm. Cytokine and neurotrophin concentrations were calculated from standard curves and normalized to total protein content.

### Statistical Analysis

Cytotoxicity was analyzed using one-way ANOVA and Tukey’s post-hoc test. The results of the BD-8 open field assay were evaluated using the Shapiro-Wilk test and ANOVA. The effects of BD-8 on LPS-induced behavioral and biochemical responses were analyzed using one-way ANOVA and Tukey’s multiple comparisons test. *p* < 0.05. Six to eight animals were included in each group. Figures and graphs are presented as mean ± standard error. *p* < 0.05 was considered statistically significant. Data analysis was performed using Prism 9 (GraphPad Software, San Diego, California, USA).

## Results

### Cell Cytotoxicity

**Fig. 1:**
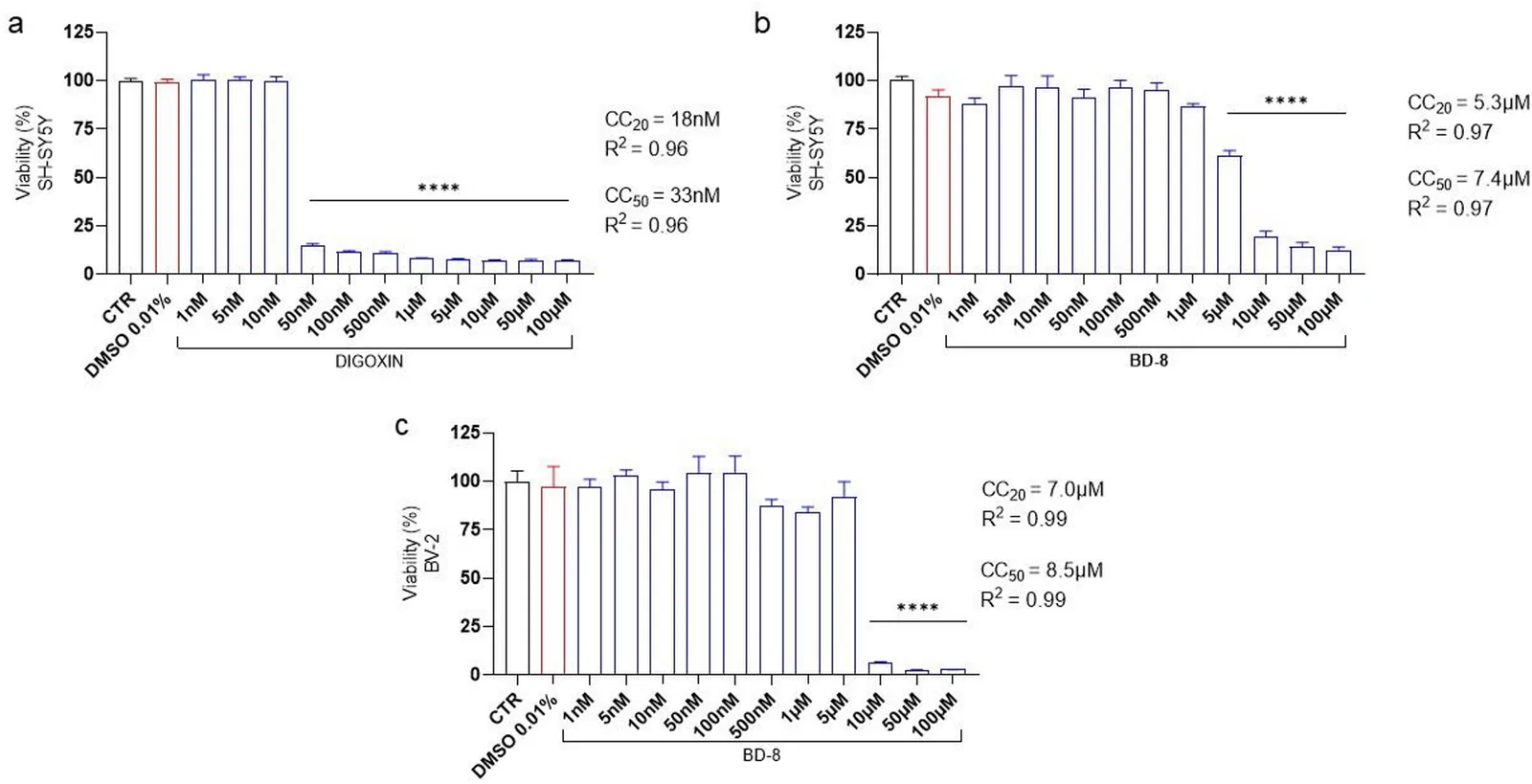
Assessment of cytotoxicity of BD-8 and digoxin in SH-SY5Y and BV-2 cells. (**a**) SH-SY5Y cells were treated with digoxin for 120h, and viability was determined using MTT assay. The CC50 and CC20 were 33 nM and 18 nM, respectively. (**b**) treatment of SH-SY5Y cells with BD-8 for 120h exhibited CC50 and CC20 7.4 μM and 5.3 μM, respectively. (**c**) BV-2 cells were treated with BD-8 for 120h, and viability was assessed using MTT assay. The CC50 and CC20 were at 8.5 μM and 7.0 μM, respectively. Data are presented as mean ± standard error of the mean (± SEM) and were analyzed by one-way ANOVA followed by Tukey’s post hoc test. **** p < 0.0001

To evaluate the cytotoxic effects of BD-8 and digoxin, we determined the CC₅₀ and CC₂₀ values in BV-2 and SH-SY5Y cells across a range of drug concentrations during 120 h. As shown in Fig. 1a, digoxin treatment in SH-SY5Y cells resulted in CC₅₀ and CC₂₀ values of 33 nM and 18 nM, respectively. In contrast, BD-8 treatment in SH-SY5Y cells (Fig. 1b) yielded markedly higher CC₅₀ and CC₂₀ values (7.4 µM and 5.3 µM, respectively), For SH-SY5Y cells treated with BD-8 (Fig. 1b), the CC₅₀ and CC₂₀ values were 7.4 µM and 5.3 µM, respectively. In BV-2 cells (Fig. 1c), BD-8 exhibited CC₅₀ and CC₂₀ values of 8.5 µM and 7.0 µM, respectively.

### BD8 Exerted an Increased Effect on Total Exploration in the Open Field Test

**Fig. 2:**
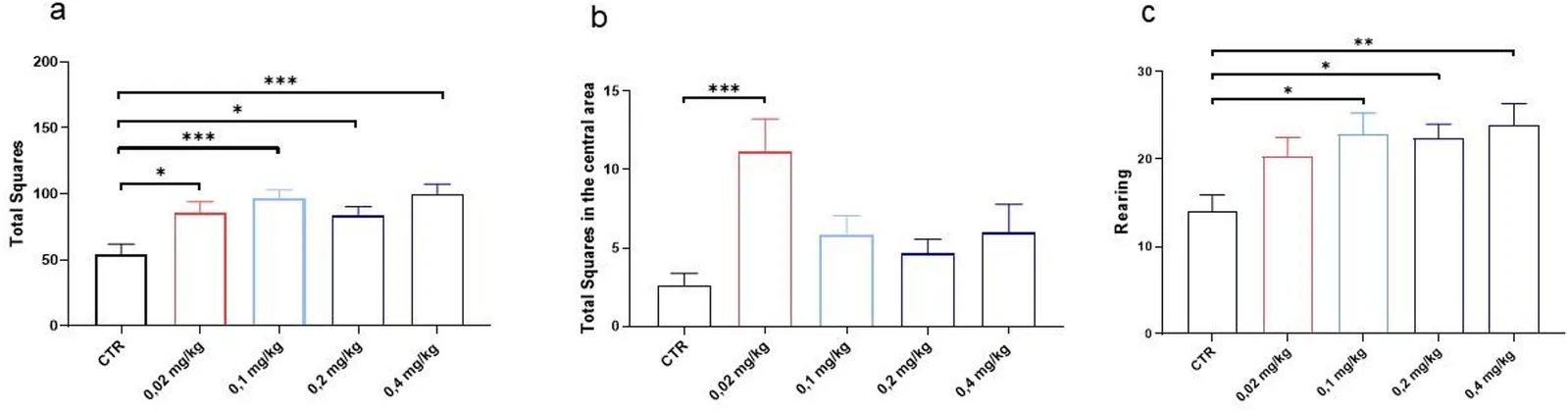
Behavioral parameters of the open field test in rats treated with BD-8. (**a)** Total squares changes (**b**) Central squares changes (**c**) Total Rearings. The results were subjected to the Shapiro-Wilk test. followed by the test of analysis of variance (ANOVA), a significance level of p<0.05 was adopted for *, p < 0,01 was adopted for **, p < 0,001 was adopted for *** n = 12

During a 3-day treatment with applications every 24 h, our results demonstrated a significant increase in the animals’ overall exploratory activity. All tested doses, 20 µg/kg (85.50 ± 8.54), 100 µg/kg (96.77 ± 6.21), 200 µg/kg (83.38 ± 6.90), and 400 µg/kg (99.75 ± 7.59), led to a higher number of total squares crossed compared to the control group (53.92 ± 8.05 squares), indicating a clear interest in exploring the new environment of the apparatus (Fig. 2a). This exploratory behavior was reinforced by the analysis of the central squares, where the 20 µg/kg to showed a significantly greater presence in the center of the arena (10.36 ± 2.08) than the control group (2.41 ± 0.7) (Fig. 2b). This finding not only corroborates the increase in exploration but also suggests a reduction in the anxiety-like state, as the animals ventured more into the exposed area of the field. Additionally, the rearing behavior, also showed a statistically significant increase at the 100 µg/kg (23.23 ± 2.60), 200 µg/kg (22.38 ± 1.62), and 400 µg/kg (23.85 ± 2.51) doses when compared to the control group (14.0 ± 1.91) (Fig. 2c). Taken together, the increase in horizontal locomotion, the greater presence in the center of the arena, and the higher frequency of rearings indicate that the treatment promoted robust exploratory behavior and an suggestive anxiolytic effect, consistent with decreased avoidance of the center.

### BD8 Presents a Systemic Biological Safety Effect in the Blood Markers

To evaluated the systemic safety of BD8, we analyzed key biochemical parameters in blood samples to identify potential deleterious effects. Our results, presented in Table 1, show that BD8 did not cause significant alterations in any of the tested markers for cardiac, liver and renal function. This finding is particularly noteworthy when contrasted with cardenolides, such as Oleandrin, which is known to induce cardiotoxicity. Previous, studies such as Botelho et al. (2020) [37] have reported that Oleandrin is associated with elevated levels of Lactate Dehydrogenase (LDH), a well-established marker of cellular damage and cardiac stress. In contrast, the absence of such changes following BD8 administration reinforces its favorable systemic safety profile, distinguishing it from other compounds with similar mechanisms of action but known toxic effects.

**Table 1:** Blood parameters analyzed in the plasma of Wistar Rats

Parameters	CTR	20 µg/Kg	100 µg/Kg	200 µg/Kg	400 µg/Kg
Total proteins (g/dL)	5.73 ± 1.19	5.94 ± 1.52	5.47 ± 1.26	6.04 ± 1.10	5.81 ± 1.28
Glucose (mg/dL)	115.75 ± 7.43	128.33 ± 7.58	116.91 ± 4.28	135.82 ± 9.68	99.73 ± 10.78
Total Cholesterol (mg/dL)	117.91 ± 14.69	110.34 ± 20.23	103.51 ± 9.32	111.35 ± 12.90	112.18 ± 11.23
Triglycerides (mg/dL)	128.60 ± 14.32	124.46 ± 12.58	116.09 ± 9.53	138.80 ± 8.98	115.86 ± 21.83
Lactate (mg/dL)	29.04 ± 7.16	25.92 ± 5.88	27.01 ± 6.85	30.07 ± 5.00	23.04 ± 5.88
Total bilirrubin (mg/dL)	0.04 ± 0.02	0.05 ± 0.02	0.03 ± 0.02	0.04 ± 0.02	0.03 ± 0.02
AST (U/L)	77.40 ± 11.38	99.65 ± 18.38	86.03 ± 12.50	90.52 ± 12.46	81.75 ± 11.94
ALT (U/L)	30.26 ± 4.08	32.50 ± 5.86	32.41 ± 4.50	29.12 ± 4.01	33.83 ± 0.82
LDH (U/L)	540.50 ± 190.56	727.20 ± 130.77	591.54 ± 183.87	1062 ± 182.44	691.28 ± 139.43
CK-MB (U/L)	147.00 ± 34.27	241.63 ± 43.33	95.25 ± 7.27	128.60 ± 16.24	173.00 ± 14.31

### BD8 prevents LPS-Induced Impairment of Locomotor Behavior

**Fig. 3:**
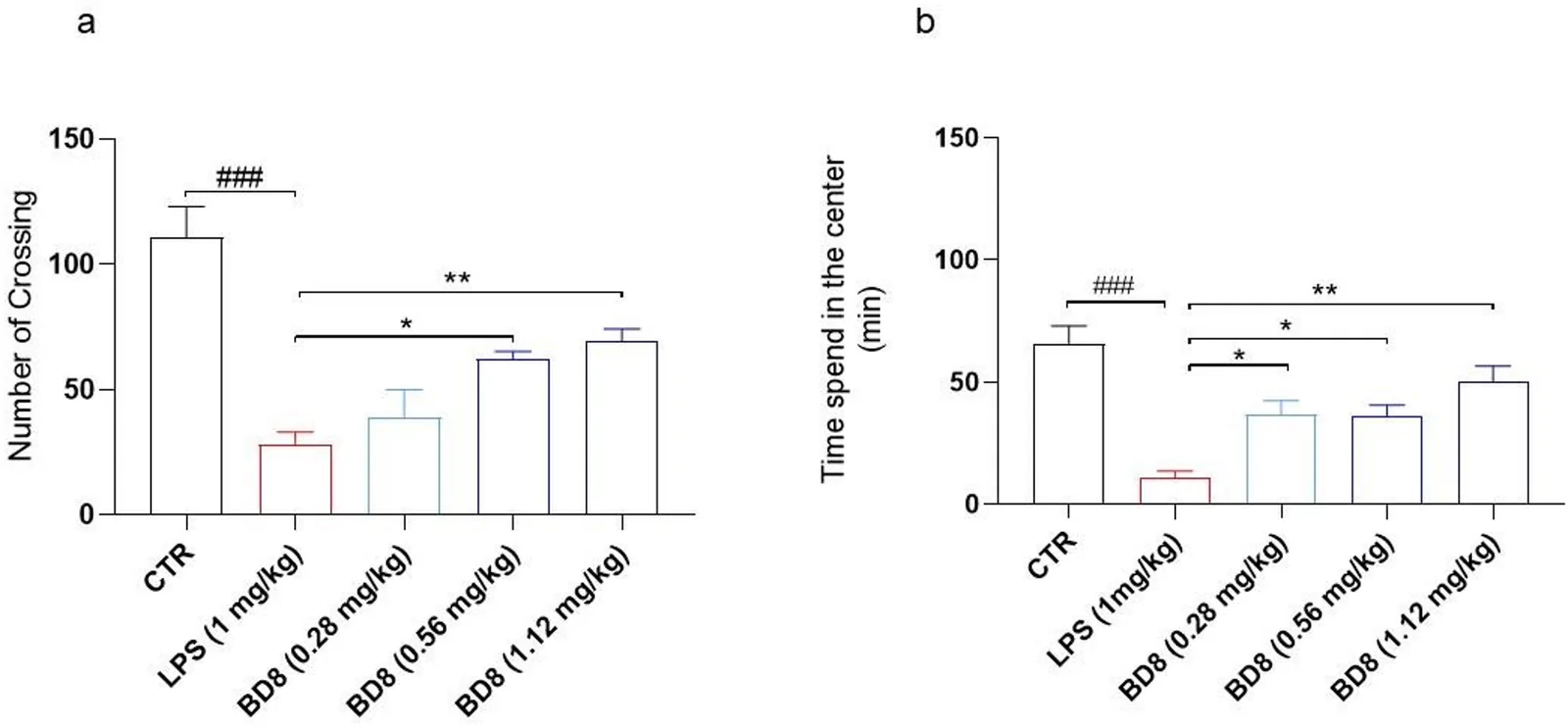
BD8 treatment reduces LPS-induced locomotor impairment in mice. (**a**) Open field test (number of crossings) and (**b**) **(**time spent in the center of the open field apparatus). Data are presented as mean ± standard error of the mean (± SEM) and were analyzed by one-way ANOVA followed by Tukey’s post hoc test. *P* < 0.05. *n* = 6–7 animals per group

Following our previous studies using LPS as a model of neuroinflammation [38], we evaluated the effect of acute BD8 pretreatment on locomotor activity in the open-field test. After a total treatment period of 4 h, our results showed that the LPS group (110.7 ± 5.094; *P* < 0.001; quadrant crossings) exhibited a significant 74.77% reduction in locomotor activity compared to the control group (27.93 ± 12.35), according to total crossing parameters (Fig. 3a). In contrast, the BD8-treated groups at doses of 0.56 mg/kg (62.07 ± 3.269; *P* < 0.05) and 1.12 mg/kg (66.31 ± 5.282; *P* < 0.001) showed a higher number of crossings compared to the LPS group (Fig. 3a). Moreover, the LPS group displayed an 83% reduction in the number of entries into the central quadrants relative to the control (CTR: 65.62 ± 7.422; LPS: 10.91 ± 2.535; *P* < 0.001), indicating probable motor dysfunction and sickness-like behavior (Fig. 3b). This effect was prevented by BD8 pretreatment at doses of 0.28 mg/kg (36.38 ± 6.078; *P* < 0.05), 0.56 mg/kg (35.98 ± 4.601; *P* < 0.05), and 1.12 mg/kg (50.10 ± 6.474; *P* < 0.01), as evidenced by an increase in time spent in the center of the open field apparatus by 233%, 229%, and 359%, respectively, compared to the LPS group (Fig. 3b).

### BD8 Reduces Lipid Peroxidation (LPO) Levels

**Fig. 4:**
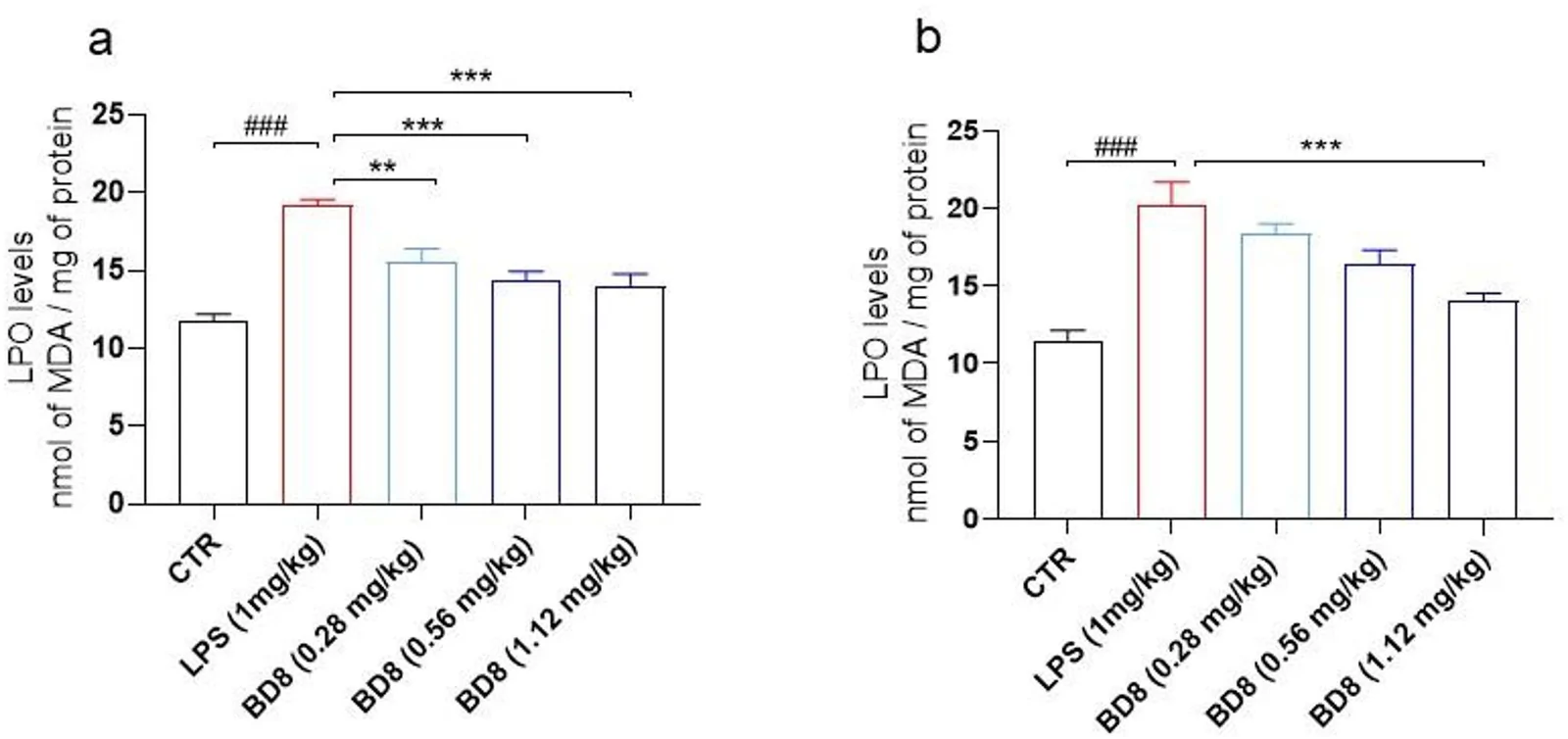
BD8 reduces LPS-induced oxidative stress in mice. (**a–b**) LPO levels in the total cortex and hippocampus. (**a**) LPO levels (nmol MDA/mg protein) in the total cortex and (**b**) LPO levels (nmol MDA/mg protein) in the hippocampus. Data are presented as mean ± standard error of the mean (± SEM) and were analyzed by one-way ANOVA followed by Tukey’s post hoc test. *P* < 0.05. *n* = 6–8 animals per group

We analyzed oxidative stress markers in the total cortex and hippocampus of mice challenged with LPS by measuring lipid peroxidation (LPO) through thiobarbituric acid-reactive substances (TBARS) levels (Fig. 4). Our data showed that LPS challenge increased LPO levels in the total cortex (19.13 ± 0.4437) (Fig. 4a) and hippocampus (20.15 ± 1.553) (Fig. 4b) 24 h after LPS administration, compared to the control group, by 63.1% and 76.3%, respectively. LPO levels in the total cortex were markedly reduced in BD8-treated groups at doses of 0.28 mg/kg (15.51 ± 0.8751; *P* < 0.01), 0.56 mg/kg (14.38 ± 0.5568; *P* < 0.001), and 1.12 mg/kg (13.94 ± 0.8372; *P* < 0.001) compared to the LPS group (Fig. 4a). In the hippocampus, however, only the 1.12 mg/kg dose (14.04 ± 0.5223; *P* < 0.001) significantly reduced LPO levels 24 h after LPS challenge (Fig. 4b).

#### BD8 Modulates Antioxidant Enzyme Activities

**Fig. 5:**
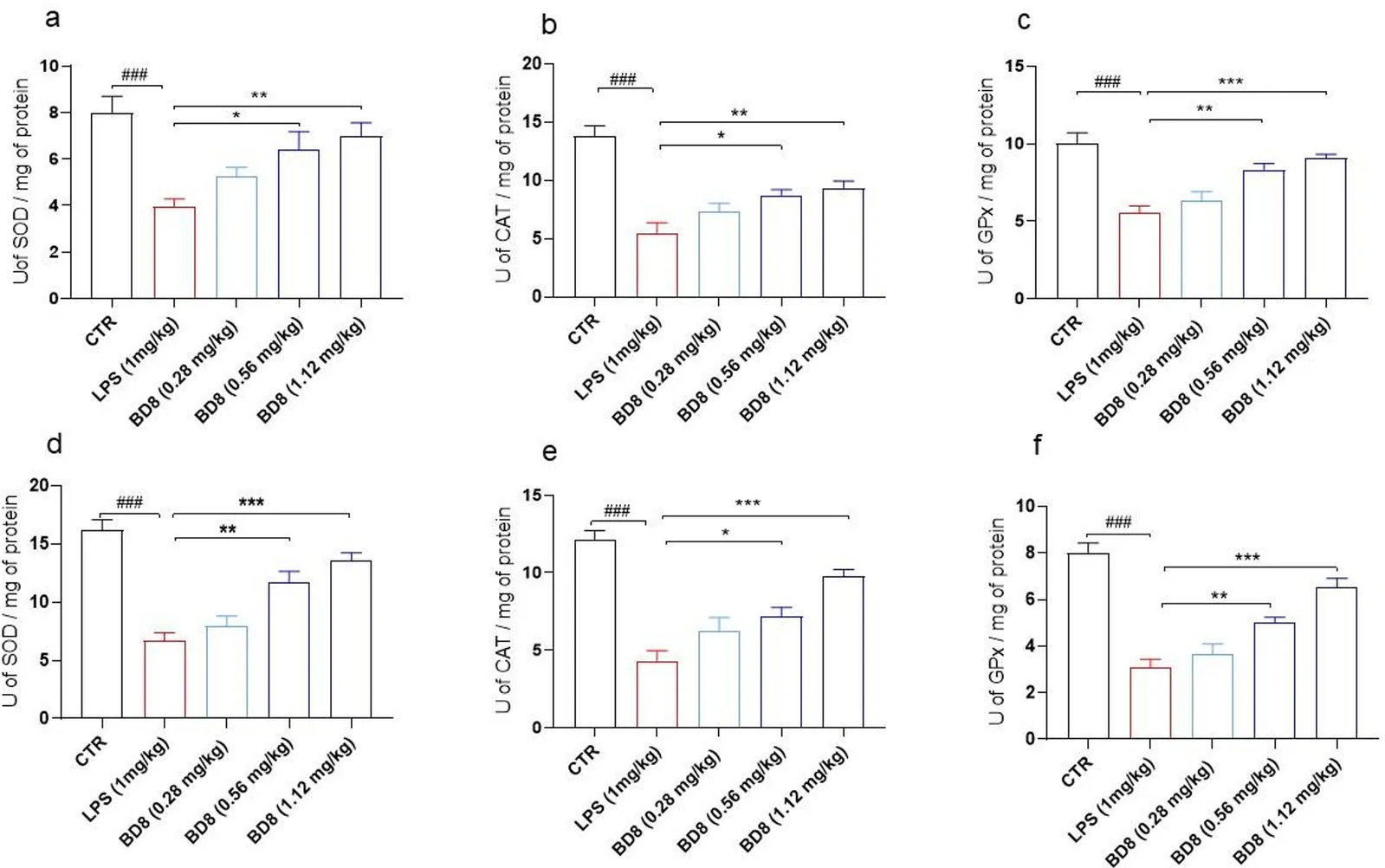
BD8 modulates antioxidant enzymes in the total cortex and hippocampus of mice after LPS challenge. (**a–c**) Total cortex; (**d–f**) Hippocampus. (**a**) and (**d**) SOD activity. (**b**) and (**e**) CAT activity. (**c**) and (**f**). GPx activity. Data are presented as mean ± standard error of the mean (± SEM) and were analyzed by one-way ANOVA followed by Tukey’s post hoc test. *P* < 0.05. *n* = 6–8 animals per group

Concomitantly, the effects of BD8 on the antioxidant defense system were evaluated in the total cortex and hippocampus of rodents after LPS challenge (Fig. 5). In the total cortex, SOD activity was significantly increased in the BD8-treated groups at 0.56 mg/kg (8.000 ± 0.7027; *P* < 0.05) and 1.12 mg/kg (7.004 ± 0.5575; *P* < 0.01) compared to the LPS group (Fig. 5a). Similarly, CAT activity was significantly elevated at doses of 0.56 mg/kg (8.711 ± 0.5037; *P* < 0.05) and 1.12 mg/kg (8.746 ± 0.6746; *P* < 0.01) relative to the LPS group (Fig. 5b), and a similar pattern was observed for GPx activity at 0.56 mg/kg (8.296 ± 0.4234; *P* < 0.05) and 1.12 mg/kg (9.072 ± 0.2476; *P* < 0.01) compared to LPS (Fig. 5c). Comparable effects were observed in the hippocampus following the LPS challenge. SOD activity was significantly increased in the 0.56 mg/kg (11.68 ± 0.9774 *P* < 0.01), and 1.12 mg/kg (13.52 ± 0.7114; *P* < 0.001) BD8-treated groups compared to LPS (Fig. 5d). Likewise, CAT activity was significantly elevated at 0.56 mg/kg (7.196 ± 0.5668; *P* < 0.05) and 1.12 mg/kg (9.787 ± 0.4146; *P* < 0.001) relative to LPS (Fig. 5e). Similar results were observed for GPx activity at 0.56 mg/kg (4.990 ± 0.2508; *P* < 0.05) and 1.12 mg/kg (6.506 ± 0.4093; *P* < 0.01) compared to LPS (Fig. 5f).

### Effects of BD-8 on mice brain IL-6 and BDNF protein levels

**Fig. 6:**
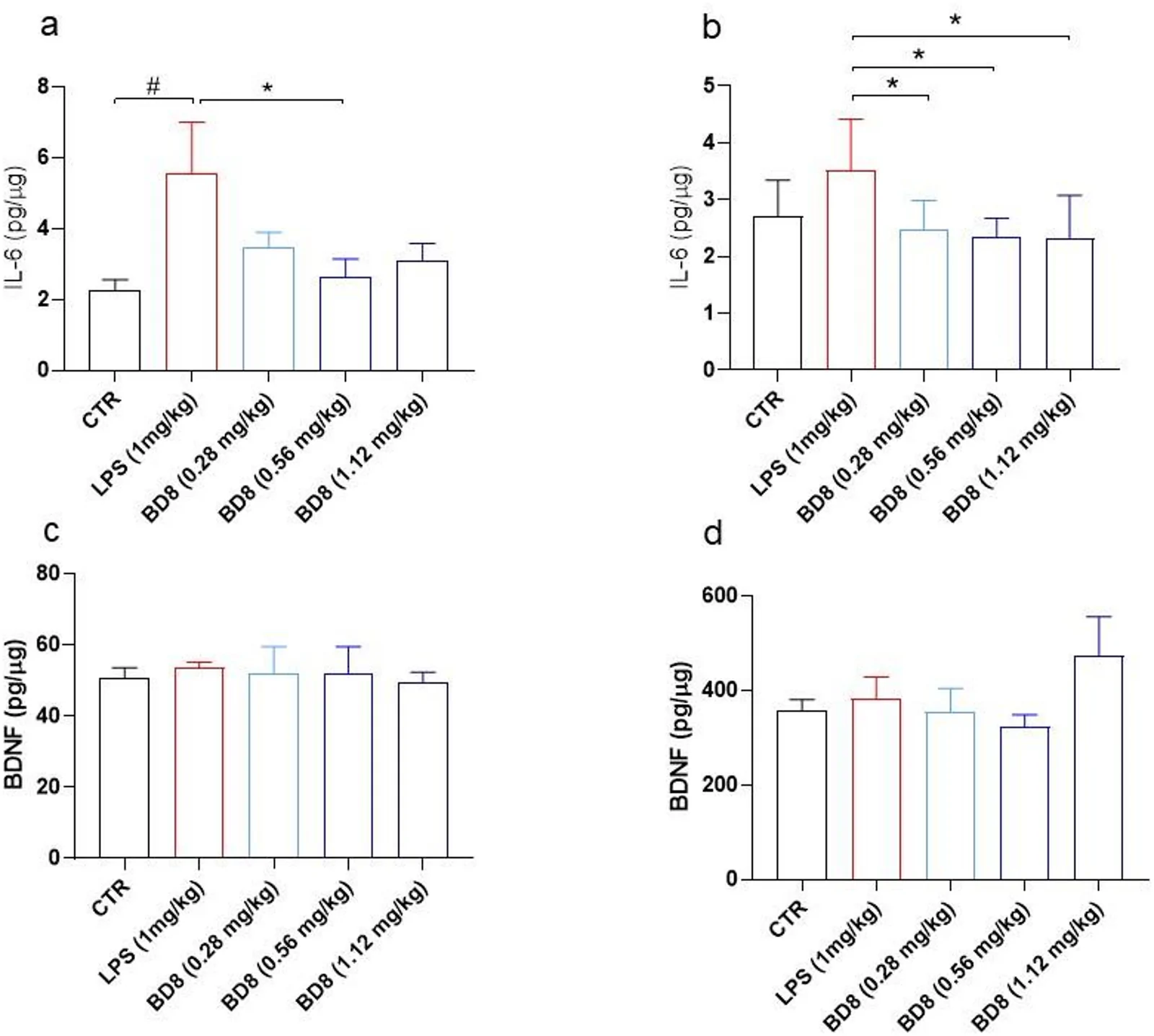
BD8 modulates IL-6 in the total cortex and hippocampus of mice after LPS challenge. (**a**) and (**c**). Total cortex; **(b)** and **(d).** Hippocampus. (**a**) and (**b**). IL-6 (**c**) and (**d**) BDNF. Data are presented as mean ± standard error of the mean (± SEM) and were analyzed by one-way ANOVA followed by Tukey’s post hoc test. *P* < 0.05. *n* = 6–8 animals per group

To evaluate the impact of BD-8 on neuroinflammation and neurotrophic support, we measured the protein levels of IL-6 and BDNF. Interestingly, treatment with BD-8 at a dose of 0.56 mg/kg significantly reduced IL-6 levels in the total cortex (2.619 ± 0.5267; *P* < 0.05) compared to the LPS group (Fig. 6a). Furthermore, doses of 0.56 mg/kg (2.333 ± 0.1193; *P* < 0.05) and 1.12 mg/kg (2.325 ± 0.2662; *P* < 0.05) reduced IL-6 levels in the hippocampus when compared to the LPS group (Fig. 6c), suggesting a protective anti-inflammatory effect. In contrast, BDNF protein expression remained unchanged across all experimental groups at the time point evaluated (Fig. 6c and d).

## Discussion

The presence of chronic neuroinflammatory processes underlies several neuropsychiatric and neurological disorders, including neurodegenerative diseases, epilepsy, and cognitive impairments [39, 40]. The glial cells’ hyperactivation occurs as part of this process, leading to the release of pro-inflammatory cytokines, oxidative stress, and progressive neuronal damage [40]. In this context, new therapeutic compounds that can mitigate inflammation in the central nervous system (CNS) are highly relevant. It is well known that cardiotonic steroids, such as ouabain and digoxin, inhibit different α-isoforms of the Na⁺/K⁺-ATPase at high concentrations. The clinical use as a cardiotonic is limited by high toxicity and a narrow therapeutic margin [41]. Furthermore, the wide distribution of NKA in tissues contributes to its systemic effects, inducing cell toxicity [22, 42]. However, several studies have shown that low concentrations of cardiotonic steroids, are linked to the activation of signaling cascades [22, 42–44]. This action has been associated with the modulation of LPS-induced glial activation, and may contribute to the attenuation of neuroinflammatory and apoptotic processes in the peripheral and central nervous system [13, 23, 39, 45].

Recent studies from our group showed that BD-8, a derivative of the cardiosteroid digoxin, exhibits high selectivity for the for the Na⁺/K⁺-ATPase α2 isoform, which is expressed in peripheral tissues (such as cardiac, muscle, and adipose cells) and in astrocytes [30, 31]. Previous data also indicated that BD-8 showed reduced neurotoxicity in in silico studies [31]. Consistently, in vitro treatment with BD-8 and Digoxin in SH-SY5Y and BV-2 cells revealed that BD-8 displays signicantly lower cytotoxicity than digoxin. Moreover, the BD-8 molecule showed a higher CC_20_ value than digoxin [31] (Ferreira et al., 2024). This data reinforces our previously work that demonstrated the role in decrease toxicity through the synthetic modification on the lactone ring [46].

Digoxin crosses the blood-brain barrier and can induce neuropsychiatric effects even at therapeutic doses [47, 48], in addition to causing neurotoxicity in animal models [49], and activating human astrocytes in vitro [50]. Previous studies demonstrate that the administration of cardiac glycosides in mice influences locomotor activity. For example, digoxin administered intragastrically at doses ≥ 1000 µg/kg significantly reduced spontaneous locomotor activity [51]. Furthermore, other studies have shown that mice treated with 650 µg/kg of digoxin exhibited a significant reduction in total distance traveled over 20 min in the open-field test, whereas doses of 1, 4, or 65 µg/kg produced no significant effect compared to the control group [52]. In contrast, our results demonstrate that a 3-day treatment with BD8, at doses of 20, 40, 200, and 400 µg/kg, promoted an increase in the animals’ locomotor and exploratory activity relative to the control group, a profile distinct from that observed with digoxin.

Treatments with cardiotonic steroids, such as Digoxin, Ouabain, and Oleandrin, are known to induce alterations in plasma biomarkers [37], making the evaluation of potential effects of BD8 treatment on cardiac, hepatic, and tissue stress markers essential. Previous studies have shown that certain concentrations of Oleandrin can significantly increase Lactate Dehydrogenase (LDH) levels, potentially suggesting cardiac toxicity, while Ouabain and Digoxin can modulate oxidative stress parameters [37]. Additionally, Ouabain has been shown to reduce markers such as LDH, ALT, and AST in leukemia induction models [53], reinforcing the need for plasma analysis to detect systemic alterations and toxic effects of cardiotonic steroids. In this context, to investigate the safety of BD8, specific biomarkers were analyzed. For hepatic function, AST and ALT were evaluated, as their elevations can indicate necrosis or inflammation [54], and total bilirubin was also evaluated, which may signal hemolysis or hepatitis [55]. Cardiac markers included CK-MB and LDH, which can indicate cardiac tissue damage [56]. Furthermore, metabolic markers of tissue stress were assessed, including cholesterol, given its direct association with cardiovascular risk; lactate, a critical indicator of tissue hypoxia; and glucose, whose elevated levels can signal acute metabolic stress [57]. The combined results demonstrate that BD8 does not alter the tested plasma biomarkers, reinforcing a favorable safety profile for the treatment.

Given the classic effects of cardiotonic steroids, such as digoxin, on cardiac tissue [58], and to further characterize the safety profile of BD-8, we investigated it effects on cardiac NKA activity and oxidative stress parameters, hydrogen peroxide and lipid peroxidation (Supplementary Fig. S5). Cardiac tissue was selected due to the high expression of the alpha 2 isoform of NKA, the primary target of BD-8, in the heart [30, 31]. Furthermore, the analysis of oxidative stress markers was supported by previous studies demonstrating that another digoxin derivative, 21-BD, can modulate parameters such as LPO, GSH, and SOD activity [59]. Our findings demonstrate that BD-8 did not induce deleterious changes in cardiac NKA activity or oxidative stress markers (Supplementary Fig. S5). These results corroborate observations from another derivative, BD-15, which also showed no harmful effects on cardiac tissue [60]. These findings are particularly relevant considering the narrow therapeutic index of digoxin (0.8–2 ng/mL) [61] and the systemic and neuropsychiatric toxicities associated with the conventional drug.

LPS remains one of the most widely used models to investigate mechanisms and therapies to decipher systemic and cerebral inflammation [62]. Peripheral administration of LPS in animals leads to a biphasic response in both behavioral and biochemical parameters. The acute phase of sickness behavior peaks within 3 to 4 h following LPS administration and manifests as a constellation of symptoms, including anhedonia, slowed movement initiation, reduced mobility, exploration, and grooming, hunched posture [63, 64]. In the present study, LPS significantly reduced locomotor activity four hours after administration, consistent with sickness behavior and anxiety- or depression-like phenotypes described in the literature [65]. Importantly, BD8 attenuated these behavioral impairments, both 0.56 mg/kg and 1.12 mg/kg increased locomotion and time spent in the center of the open field, suggesting partial attenuation of sickness-related behavioral alterations. Locomotion is frequently impaired in neurological conditions such as Parkinson’s disease, stroke, multiple sclerosis, and dementia [66]. These neurodegenerative diseases are associated with the aging process, which is also related to increased basal levels of oxidative stress and neuroinflammation mediators [67, 68].

Oxidative stress arises when the production of reactive oxygen species (ROS) exceeds the antioxidant defense capacity, leading to damage to lipids, proteins, and DNA. The brain is particularly vulnerable to this imbalance due to its high oxygen consumption and relatively low antioxidant reserves [69]. Furthermore, the interaction of cardiotonics with Na⁺/K⁺-ATPase can generate ROS by modulating cellular signaling pathways [70], a process relevant in conditions such as obesity and cardiovascular disease [71]. Oxidative stress plays a central role in the behavioral changes induced by LPS, with lipid peroxidation among the most consistent markers of this response. It has already been shown that LPS increases oxidative stress and damage to the prefrontal cortex and hippocampus in Parkinson’s and Huntington’s disease models [72], in addition to being associated with depression- and anxiety-like behaviors [73]. Corroborating these findings, we observed that LPS significantly increased LPO levels in the total cortex and hippocampus in our model. Previous results from our group have shown that Ouabain and BD-15 reduce LPS-induced lipid peroxidation in multiple brain structures [24, 28, 74]. Furthermore, in vitro studies have shown that digoxin reduces LPO levels [59]. In this work, we show that BD8 also reduces lipid peroxidation in the total cortex and hippocampus after LPS challenge, reinforcing the potential of digoxin-derived compounds to modulate oxidative stress in neuroinflammatory conditions.

Moreover, LPS administration has been shown to decrease the activity of key antioxidant enzymes, including GPx, SOD, and CAT, particularly in the prefrontal cortex and hippocampus [75]. Here, BD8 effectively reversed these alterations, restoring the activity of these enzymes in the total cortex and hippocampus. Supporting these findings, other studies have demonstrated that digoxin-derived compounds, such as BD-15, also modulate antioxidant enzyme activity in LPS-induced neuroinflammation [28], and that ouabain exerts regulatory effects on antioxidant defenses [24].

Interleukin-6 (IL-6) has been recognized as an important mediator of the neuroinflammatory response induced by LPS, being associated with fever, sickness behavior, and motivational impairments [76–78]. Previous studies demonstrated that neutralization of IL-6 attenuates LPS-induced fever, supporting the involvement of this cytokine in inflammatory responses [79]. In addition, increased brain IL-6 levels have been associated with cognitive impairment [80]. In the present study, BD-8 treatment was associated with reduced IL-6 levels in the total cortex and hippocampus after LPS administration, suggesting a modulatory effect of this compound on the neuroinflammatory response. Considering that elevated IL-6 levels have also been related to motor impairments in inflammatory models [65, 81], it is possible that the reduction observed in BD-8-treated animals may have contributed, at least in part, to the improved motor performance observed in our study. In agreement with this interpretation, previous reports have shown that interventions capable of reducing IL-6 levels are frequently associated with better motor outcomes [82]. Interestingly, no significant changes were observed in BDNF levels, suggesting that the effects of BD-8 in our model may be more closely related to the modulation of inflammatory pathways rather than alterations in neurotrophic signaling. In contrast to our findings, other cardiotonic steroids, such as Ouabain and oleandrin, have been reported to increase BDNF levels [23, 44] indicating that distinct compounds within this class may differentially regulate neurotrophic pathways.), which may indicate a shared characteristic of this class of compounds.

**Fig. 7:**
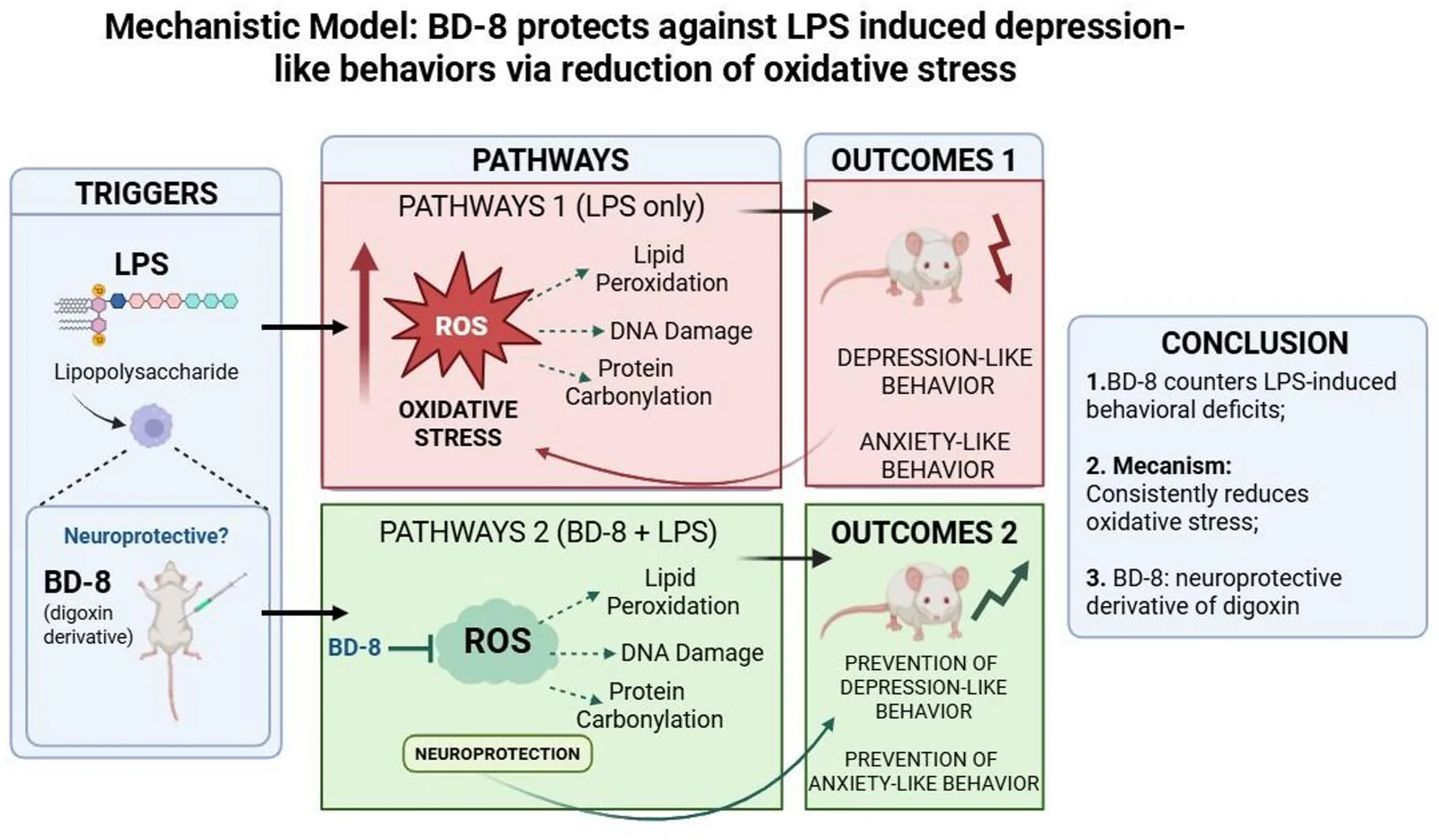
Schematic representation of the protective effects of BD8 on LPS-induced neurobehavioral alterations through modulation of inflammatory and oxidative stress

Mechanistic overview supported by measured endpoints is illustrated in Fig. 7. LPS administration resulted in decreased locomotor activity, and BD8 treatment attenuated this behavioral alteration in mice challenged with LPS. These behavioral effects were accompanied by modulation of inflammatory and oxidative stress-related parameters, including reduced IL-6 levels and changes in antioxidant enzyme activity.

## Conclusion

Taken together, the results indicate that the effects of BD-8 on LPS-induced neurobehavioral. The behavioral improvements were accompanied by modulation of inflammatory and oxidative stress-related parameters, including reduced IL-6 levels and alterations in antioxidant enzyme activity, supporting BD-8 as a promising candidate for neuroinflammatory disorders.

## Supplementary Information

Below is the link to the electronic supplementary material.

Supplementary Material 1

## Data Availability

The data that support the findings of this study are available from the corresponding author upon reasonable request.
