# Supplementary material for: Digoxin Derivative BD-8 Attenuates Oxidative Stress and Neuroinflammation in an LPS-Induced Experimental Model
Source: Cell Biochem Biophys. 2026 Jun 25;84(3):4131–45. doi: 10.1007/s12013-026-02108-x (PMC13586031; doi:10.1007/s12013-026-02108-x)
Supplement: Supplementary file 1 [file 12013_2026_2108_MOESM1_ESM.docx]

**Supplementary Information**

**Digoxin derivative BD-8 attenuates oxidative stress and neuroinflammation in an LPS-induced experimental model**

**Vitória Karolina Brandão Souza^1a^, Davi Azevedo Ferreira^2a^, Antônio Pereira Ribeiro Arantes^3^, Elton de Sá Figueiredo^2^, Millena Ferreira Rodrigues Fonseca^1^, Pâmela Yasmin de Oliveira Ferreira^1^, Martina Raissa Ribeiro^5^; Paulo César Ghedini^1^, Andreza Amália de Freitas Ribeiro^4^, José Augusto Ferreira Perez Villar^4^, Maira de Castro Lima^3^, Israel José Pereira Garcia^3^; Hérica L Santos^3^; Cristoforo Scavone^5^, Sandra Rodrigues Mascarenhas^2^, Jacqueline Alves Leite^1*^**

^1^Instituto de Ciências Biológicas, Department of Pharmacology, Federal University of Goiás, Goiânia, GO, Brazil, Zip Code: 74690-612

^2^Laboratory of Immunopharmacology, Biotechnology Center, Federal University of Paraíba, João Pessoa, PB, Brazil, Zip Code: 58.051-900.

^3^Laboratório de Bioquímica Celular, Campus Centro-Oeste Dona Lindú, Federal University of São João del-Rei, Divinópolis, MG, Brazil, Zip Code: 35.501-296.

^4^Laboratório de Síntese Orgânica e Nanoestruturas – Campus Centro-Oeste Dona Lindú, Federal University of São João del-Rei, Divinópolis, MG, Brazil, Zip Code: 35.501-296.

^5^Neuropharmacology Research Laboratory, University of São Paulo, SP, Brazil, Zipe Code: 05508-000.

*Correspondence: Jacqueline Alves Leite, Email: jacquelineleite@ufg.br

^a^The first two authors share equal first authorship.

**Materials and Methods**

**Instrumental and Analytical Characterization**

All NMR data were acquired in CDCl_3_ on Bruker BioSpin Avance NEO 600 MHz  (UFMG-DQ). The spectrometer was equipped with a 5-mm multinuclear direct detection probe with z-gradient. All ^1^H- and ^13^C-NMR chemical shifts are given in ppm (*δ*) relative to the TMS signal at 0.00 ppm and the coupling constants (*J*) are in Hz. HPLC analysis was performed in a Shimadzu Prominence liquid chromatography system, equipped with a LC-20AT pump, SPD-M20A Photodiode Array detector, SIL-20A automatic injector, and a Shim-pack C-18 VP-ODS 4.6 × 250 mm, 5 μm reverse-phase column.

ESI-MS analysis were obtained using Amazon SL, Bruker Daltonics spectrometer (Central Analítica LCACCO-UFSJ), operated by direct infusion through an electrospray source in negative mode resulting in [M-H]^-^ ions.

A HRMS analysis were obtained using anq-ToF Maxis 3G, Bruker Daltonics spectrometer (Central Analítica IQ-USP), operated by direct infusion through an electrospray source in both positive modesresulting in [M+H]^+^ and [M+Na]^+^ ions.

**Na,K-ATPase activity**

The Na,K-ATPase assay was performed in 500 μL conical tubes with 10 μg of membrane fraction protein in a reaction medium containing 40mM Tris-HCl (pH 7.4), 120mM NaCl, 20 mM KCl, 5 mM MgCl2, and 3 mM ATP, with or without ouabain (10 μM or 1 mM). After a one-hour incubation at 37°C, the reaction was stopped by adding 1% SDS, and 100 μL of Fiske solution was added to complex the inorganic phosphate released from ATP hydrolysis. The samples were then transferred to a 96-well plate for spectrophotometric reading at 660 nm. Total Na,K-ATPase activity was calculated by subtracting the activity obtained with 1 mM ouabain from the activity without ouabain (inhibiting α1, α2, and α3). All experiments were conducted in triplicate, and results were expressed as nmol Pi/min/mg protein [1].

**Hydrogen Peroxide production**

H_2_O_2_ quantification was performed by adding 100 μL of homogenate to 900 μL of reaction medium (250 μM ferrous ammonium sulfate, 100 μM xylenol orange, and 25 mM H2SO4). After vortexing and 45 minutes of light protection (due to H2O2 photosensitivity), readings were taken using a spectrophotometer (Genesys 10 uv scanning, Thermo Scientific®) at 580 nm. H_2_O_2_ production was calculated based on a standard curve from 0 to 100 nM of PA H2O2 [2].

**Lipid peroxidation (LPO) levels**

The LPO levels quantification was performed using a colorimetric thiobarbituric acid assay, adapted from Buege and Aust (1978). For this, 60 μL of homogenate, 200 μL of pH 7.4 sodium phosphate buffer, 200 μL of 12% trichloroacetic acid, and 400 μL of 1% thiobarbituric acid were added to 1.5 mL microtubes. After vortex homogenization, the tubes were heated at 95ºC for 30 minutes and subsequently cooled in an ice bath for 10 minutes. Following this, centrifugation was performed at 10,000 g for 5 minutes. For ELISA reader measurement at 532 nm, only 215 μL of the supernatant were transferred to 96-well plates to avoid precipitate interference. MDA quantification (nmol MDA/mg protein) was determined from a standard curve [3].

**Supplementary Figure S1. Structural elucidation data**

**^1^H RMN** (600 MHz, CDCl_3_) δ (ppm): 7.29 (s, 1H), 7.24 (d, *J* = 8.3 Hz, 1H), 7.21 (d, *J* = 8.3 Hz, 1H), 6.84 – 6.79 (m, 2H), 6.69 – 6.61 (m, 4H), 4.90 (ddd, *J* = 9.8, 5.9, 2.2 Hz, 4H), 4.85 (dd, *J* = 9.6, 2.2 Hz, 2H), 4.24 (dq, *J* = 6.7, 3.3 Hz, 4H), 4.10 (q, *J* = 3.1 Hz, 4H), 4.02 (s, 2H), 3.97 (s, 1H), 3.95 – 3.88 (m, 3H), 3.83 (dd, *J* = 9.4, 6.3 Hz, 2H), 3.76 (ddd, *J* = 9.6, 6.4, 3.7 Hz, 4H), 3.59 – 3.54 (m, 1H), 3.38 (t, *J* = 8.7 Hz, 1H), 3.29 (dd, *J* = 9.5, 3.3 Hz, 1H), 3.24 (dd, *J* = 9.3, 3.0 Hz, 1H), 3.20 (dd, *J* = 9.4, 3.0 Hz, 1H), 3.09 (s, 2H), 3.03 (s, 2H), 2.19 – 2.02 (m, 11H), 1.99 – 1.90 (m, 2H), 1.86 – 1.41 (m, ~50H), 1.28 (d, *J* = 6.3 Hz, 6H), 1.23 (d, *J* = 6.2 Hz, 12H), 0.96 (t, *J* = 7.4 Hz, 6H), 0.92 (s, 3H), 0.91 (s, 3H), 0.74 (s, 3H), 0.72 (s, 3H).

**^13^C RMN** (150 MHz, CDCl_3_) δ (ppm): 9.12, 9.30, 13.86, 18.17, 19.22, 21.79, 23.52, 23.55, 26.48, 26.60, 27.67, 28.10, 29.11, 29.17, 29.78, 30.13, 31.25, 31.27, 32.23, 33.19, 35.03, 36.22, 36.69, 37.11, 37.80, 41.64, 41.72, 42.51, 42.75, 56.32, 56.40, 56.82, 56.96, 66.35, 66.46, 67.63, 67.65, 68.05, 68.09, 68.26, 69.51, 72.54, 72.67, 76.69, 76.73, 82.19, 82.55, 86.64, 95.40, 98.23, 98.29, 113.78, 114.02, 114.87, 114.97, 120.38, 120.43, 129.96, 129.99, 133.48, 133.69, 146.61, 146.81, 159.52, 159.55, 168.67, 168.87, 199.94, 200.14, 204.06, 204.17.

**HRMS** m/z calculated for C_52_H_76_O_15_ [M+Na]^+^: 963.5076, found 963.5094.

**ESI MS low resolution** m/z calculated for C_52_H_76_O_15_ [M-H]^-^: 939.51, found 939.5037; m/z calculated for C_52_H_76_O_15_ [M-Cl]^-^: 975.49, found 975.72.

**Supplementary Figure S2. ^1^H, ^13^C and DEPT-135 NMR spectra of BD-8**

**Supplementary Figure S3. High resolution mass spectra of BD-8**

**Supplementary Figure S4. ESI-MS low resolution of BD-8**

**Supplementary Figure S5**

The administration of diferentes doses of BD8 did not induce cardiotoxicity or biochemical alterations in the heart tissue. As shown in Supplementary Figure S5, Na⁺/K⁺-ATPase activity (Figure a) remained stable across all groups. Furthermore, markers of oxidative stress were not significantly affected, as evidenced by the LPO levels (Figure b) and H2O2 production levels (c) compared to the control group. Statistical analysis confirmed no significant differences (P > 0.05) between the treated and control animals.

**Supplementary Figure S5.** BD8 causes no damage to the heart. (**a**) Na⁺/K⁺-ATPase activity on heart (**b**) LPO levels (**c**) H_2_O_2_ production. Data are presented as mean ± standard error of the mean (± SEM) and were analyzed by one-way ANOVA followed by Tukey’s post hoc test. P < 0.05. n = 6 animals per group

**References**

1. Fiske C. H., Subbarow, Y. (1925). The Colorimetric Determination of Phosphorus. *Journal of Biological Chemistry*, p. 375–400, 1925.
2. Jiang, Z. Y., Woollard, A. C., & Wolff, S. P. (1990). Hydrogen peroxide production during experimental protein glycation. *FEBS letters*, *268*(1), 69–71. <https://doi.org/10.1016/0014-5793(90)80974-n>
3. Buege, J. A., & Aust, S. D. (1978). Microsomal lipid peroxidation. *Methods in enzymology*, *52*, 302–310. <https://doi.org/10.1016/s0076-6879(78)52032-6>
